# Brachial-ankle pulse wave velocity and metabolic syndrome in general population: the APAC study

**DOI:** 10.1186/s12872-016-0409-x

**Published:** 2016-11-18

**Authors:** Anxin Wang, Zhaoping Su, Xiaoxue Liu, Yuling Yang, Shuohua Chen, Suzhen Wang, Yanxia Luo, Xiuhua Guo, Xingquan Zhao, Shouling Wu

**Affiliations:** 1 Department of Neurology, Beijing Tiantan Hospital, Capital Medical University, No. 6 Tiantanxili, Dongcheng District, Beijing, 100050 People’s Republic of China; 2 China National Clinical Research Center for Neurological Diseases, Beijing, People’s Republic of China; 3 Center of Stroke, Beijing Institute for Brain Disorders, Beijing, People’s Republic of China; 4 Beijing Key Laboratory of Translational Medicine for Cerebrovascular Disease, Beijing, People’s Republic of China; 5 Department of Epidemiology and Health Statistics, School of Public Health, Capital Medical University, Beijing, 100069 People’s Republic of China; 6 Department of Epidemiology and Health Statistics, Academy of public health and management, Weifang Medical University, No. 7166 Baotongxijie, Weicheng District, Weifang, 261053 People’s Republic of China; 7 Department of Cardiology, Tangshan People’s Hospital, Tangshan, People’s Republic of China; 8 Graduate School, North China University of Science and Technology, Tangshan, People’s Republic of China; 9 Department of Cardiology, Kailuan Hospital, North China University of Science and Technology, No. 57 Xinhua Road, Lubei District, Tangshan, 063000 People’s Republic of China

**Keywords:** Brachial-ankle pulse wave velocity, Metabolic syndrome, Arterial stiffnes, Cardio-cerebrovascular diseases

## Abstract

**Background:**

Metabolic syndrome (MetS) is correlated with arterial stiffness and can be evaluated by brachial-ankle pulse wave velocity (baPWV). We investigated potential associations between MetS and baPWV in a Chinese community population.

**Methods:**

The community-based Asymptomatic Polyvascular Abnormalities in Community study examined asymptomatic polyvascular abnormalities in a Chinese population aged ≥40 years. The relationship between MetS and its components and baPWV was analyzed by multivariate logistic and linear regression models.

**Results:**

Out of 5181 study participants, 1271 subjects (24.53%) had MetS. Mean values of baPWV in subjects with 0, 1, 2,3, 4, and 5 components of MetS were 1430, 1526, 1647, 1676,1740, and 1860 cm/s, respectively (*p* < 0.001 for trend). After adjusting for confounding risk factors, MetS was significantly associated with baPWV (odds ratio [OR]: 2.74; 95% CI: 2.28, 3.30). Among the five components of MetS, elevated blood pressure was the most important factor for baPWV. All models of multivariate linear regression analysis showed a significant positive correlation between the increasing numbers of MetS components and baPWV (*p* < 0.0001).

**Conclusions:**

baPWV was associated with MetS and was greater with increasing numbers of MetS components. Elevated blood pressure was the most important factor for baPWV.

## Background

Metabolic syndrome (MetS) is a pre-atherosclerotic state which involves a cluster of factors, such as abdominal obesity, arterial hypertension, dyslipidemia, and hyperglycemia, and is closely associated with a marked increase in the risk of cardio-cerebrovascular diseases and type 2 diabetes mellitus [1–3]. Some studies have shown that MetS is correlated with arterial stiffness. Nakanishi et al. suggested that persistent MetS conditions can deteriorate the severity of arterial stiffness [4, 5]. Furthermore, Tomiyama et al. suggested that an improvement in MetS status could delay the progression of atherosclerosis [6].

Pulse wave velocity is an objective valid index of arterial stiffness and is widely used for non-invasive evaluation of subclinical atherosclerotic changes [7]. Brachial-ankle pulse wave velocity (baPWV), a type of pulse wave velocity, is a promising test method that can assess the stiffness of both aortic and peripheral arteries [8, 9]. baPWV not only reflects early atherosclerotic change [10–12], but also serves as a valuable predictor of mortality due to cardio-cerebrovascular events [13]. In the past decade, studies have investigated the relationship between arterial stiffness measured by baPWV and the components of MetS in different populations worldwide. However, the association of baPWV with MetS is not understood well. The correlation between baPWV and MetS and its components may be different based on different populations, regions and cultural diet.

The purpose of the present study was to evaluate the relationship between baPWV and MetS and its individual components in a large sample size of 5181 community-based subjects in the northern region of China, and to further elucidate the complex correlation between baPWV and MetS.

## Methods

### Study population

The Asymptomatic Polyvascular Abnormalities Community (APAC) study is a community-based observational prospective, long-term follow-up study to investigate the epidemiology of asymptomatic polyvascular abnormalities in Chinese adults [14]. The study cohort was a subgroup of a previously described population of the Kailuan study [15]. Data about patient demographics , clinical characteristics (physical examination, laboratory tests, carotid duplex ultrasound and transcranial Doppler examinations), and also finished structured interviews with a standardized questionnaire performed by trained investigators. Anthropometric indices included height and weight. Body mass index (BMI) was calculated as body weight (kg) divided by the square of body height (m^2^). Blood pressure was measured twice with an appropriate cuff size in relation to arm size, in the participants with the supine position after 10 min resting. The average of the two readings was used. However, if the difference between the two measurements exceeded 5 mmHg, a third reading would be conducted, and the average of three readings was recorded and analyzed. Smoking was defined as at least one cigarette per day for more than a year. Alcohol abuse was defined as alcohol intake of at least 90 and 45 g of liquor a day for men and women, respectively, for more than 1 year. Smoking or drinking cessation was considered only if it lasted for at least 1 year. Details of the APAC study design and the information on baseline characteristics have been published previously. The community is part of a large coal mining industry in Tangshan, Hebei Province [15, 16], China. The study protocol was approved by the Ethics Committees of the Kailuan General Hospital and the Beijing Tiantan Hospital, and informed consent was obtained from all participants.

### Definition of MetS

MetS was defined using previously published criteria from the International Diabetes Federation [11]. The definition included the presence of central obesity (waist circumference >90 cm for Chinese men and >80 cm for Chinese women), plus any two of the following factors: triglycerides >150 mg/dl (1.7 mmol/L) or obtaining therapy for increased triglyceride (TG) concentrations; high-density lipoprotein cholesterol (HDL-C), 40 mg/dl (1.03 mmol/L) in men and 50 mg/dl (1.29 mmol/L) in women, or obtaining therapy for low HDL-C level; systolic blood pressure (BP) >130 mmHg or diastolic BP >85 mmHg, or treatment of previously diagnosed arterial hypertension; fasting plasma glucose >100 mg/dl (5.6 mmol/L) or previously diagnosed type 2 diabetes mellitus.

### Measurement of baPWV

Bilateral baPWV was evaluated by utilizing aBP-203RPE III device ( Omron Healthcare, Kyoto, Japan). Details about this instrument and its use have been described, and clinical validation and good reproducibility have been confirmed [8]. All examinations were carried out by specially trained physicians and nurses. All subjects were light clothing and underwent examination under resting-state by placement of the subject in a supine position without a pillow. Electrodes of the electrocardiograph were located in both wrists, a stethoscope was located in the left border of the sternum for acquiring heart sounds, and cuffs were wrapped with certain strain on both the upper arms and ankles. The lower border of the brachial cuff was located 2–3 cm above the chelidon, and the lower border of ankle cuff was located 1–2 cm above the medial malleolus. Two measured values were recorded from both sides of the body, while the second value of baPWV was utilized. Then we used the higher of the left and right baPWV values for analyses [17].

Arterial stiffness was considered as baPWV ≥1400 cm/s, since this is what has been identified as an independent risk factor in the Framingham score and can distinguish patients with atherosclerotic cardiovascular disease. Diabetes was considered as fasting plasma glucose concentration of ≥7.0 mmol/L or treatment for diabetes with drug or insulin. Hypertension was defined as systolic BP >130 mmHg or diastolic BP >85 mmHg or on drug treatment for hypertension.

### Statistical analysis

The continuous variables and categorical data are expressed as the mean ± standard deviation (SD) and percentage, respectively. The Student’s t-test or ANOVA test was used to address non-paired samples for the comparison of normally distributed parameters, and the Wilcoxon or Kruskal-Wallis test for the comparison of non-parametric variables. The qualitative variables were compared by using chi-squared test. Four multivariate logistic regression analyses were performed to calculate odds ratio (OR) and 95% confidence interval (CI) for the associations of MetS or the number of MetS components (the 0 MetS component group was used as the reference category). Arterial stiffness was defined as baPWV ≥1400 cm/s. Model 1 was the crude model; Model 2 adjusted for age and gender; Model 3 adjusted for age, gender, level of education, income, smoking, alcohol abuse, and physical activity; and Model 4 adjusted for age, gender, level of education, income, smoking, alcohol abuse, amount of physical activity, BMI, and serum concentrations of low-density lipoprotein cholesterol (LDL-C) and high-sensitivity C-reactive protein. Finally, for each model, a trend test was performed after the number of MetS components as a continuous variable entering into the model. Furthermore, a multivariate linear regression was applied to analyze the independent associations between baPWV and MetS. Differences were considered statistically significant only when *p*-value was <0.05. All data were analyzed with a commercially available software program (SAS software version 9.3; SAS Institute Inc., Cary, NC, USA).

## Results

Of the 5440 subjects who were originally included into the APAC study, we excluded 218 participants without measurement of baPWV and 41 individuals without MetS. Eventually, 5181 participants (3108 men, 2073 women) with a mean age of 55.16 ± 11.80 years (range: 40–94 years) were included in the present investigation. The distribution of demographics, anthropometric measurement, baPWV, vascular risk factors and living habits according to presence of metabolic syndrome are summarized in Table 1. At baseline, 1271 out of 5181 subjects (24.53%) had MetS. Compared with those with no MetS, the incidence of MetS was found significantly in older subjects, in higher proportion of men, in subjects with lower level of education, subjects at the middle-income level (*p* < 0.001 for all). There were no statistically significant differences for smoking (*p* = 0.3655), drinking (*p* = 0.7074) and physical activity (*p* = 0.6289) between the MetS group and the group without MetS. The baPWV values were significantly and positively correlated with the values of the components of MetS (*p* < 0.0001).

**Table 1:** Clinical and biochemical characteristics of subjects with and without metabolic syndrome

Variables	Total (*n* = 5181)	MetS group (*n* = 1271)	Non-MetS group (*n* = 3910)	*P* value
Age (year)	55.16 ± 11.80	56.29 ± 10.47	54.79 ± 12.18	<0.0001
Male, n (%)	3108 (59.99)	701 (55.15)	2407 (61.56)	<0.0001
Education, n (%)				
Illiteracy/Primary School	638 (12.31)	188 (14.79)	450 (11.51)	0.0004
Middle School	2295 (44.30)	583 (45.87)	1712 (43.79)	
High School or Higher	2248 (43.39)	500 (39.34)	1748 (44.71)	
Income, n (%)				
< ¥1000	1239 (23.91)	264 (20.77)	975 (24.94)	0.0001
¥1000–3000	3431 (66.22)	903 (71.05)	2528 (64.65)	
> ¥3000	511 (9.86)	104 (8.18)	407 (10.41)	
Body Mass Index (kg/m2)	24.94 ± 3.25	27.38 ± 3.00	24.15 ± 2.92	<0.0001
Waist circumference (cm)	86.12 ± 9.66	93.90 ± 7.26	83.59 ± 8.96	<0.0001
Waist-to hip ratio	0.88 ± 0.06	0.91 ± 0.06	0.87 ± 0.06	<0.0001
Systolic Blood Pressure (mmHg)	131.33 ± 20.04	141.18 ± 18.36	128.12 ± 19.51	<0.0001
Diastolic Blood Pressure (mmHg)	82.86 ± 11.06	88.10 ± 10.79	81.16 ± 10.60	<0.0001
Fasting Plasma Glucose (mmol/L)	5.59 ± 1.51	6.37 ± 2.01	5.33 ± 1.20	<0.0001
Total cholesterol (mmol/L)	1.53 ± 0.70	1.69 ± 0.74	1.48 ± 0.68	<0.0001
Triglycerides (mmol/L)	1.68 ± 1.42	2.60 ± 2.01	0.38 ± 1.00	<0.0001
High-Density Lipoprotein-Cholesterol ( mmol/L)	1.63 ± 0.46	1.45 ± 0.41	1.68 ± 0.46	<0.0001
Low-Density Lipoprotein-Cholesterol (mmol/L)	2.63 ± 0.74	2.76 ± 0.87	2.58 ± 0.69	<0.0001
High-Sensitivity C-Reactive Protein	2.13 ± 4.18	2.77 ± 4.25	1.92 ± 4.14	<0.0001
bapwv (cm/s)	1588.40 ± 401.62	1703.30 ± 371.57	1551.06 ± 403.98	<0.0001
Hypertension, n (%)	2500 (48.25)	980 (77.10)	1520 (38.87)	<0.0001
Diabetes mellitus, n (%)	629 (12.14)	348 (27.38)	281 (7.19)	<0.0001
Smoking, n (%)				
Never	3233 (62.40)	812 (63.89)	2421 (61.92)	0.3655
Former	291 (5.62)	73 (5.74)	218 (5.58)	
Current	1657 (31.98)	386 (30.37)	1271 (32.51)	
Alcohol abuse, n (%)				
Never	3391 (65.51)	833 (65.54)	2561 (65.50)	0.7074
Former	81 (1.56)	23 (1.81)	58 (1.48)	
Current	1706 (32.93)	415 (32.65)	1291 (33.02)	
Physical Activity, n (%)				
Inactive	2087 (40.28)	506 (39.81)	1581 (40.43)	0.6289
Moderately Active	1312 (25.32)	314 (24.70)	998 (25.52)	
Very Active	1782 (34.39)	451 (35.48)	1331 (34.04)	

*MetS* metabolic syndrome, *baPWV* brachial-ankle pulse wave velocity

**Fig. 1:**
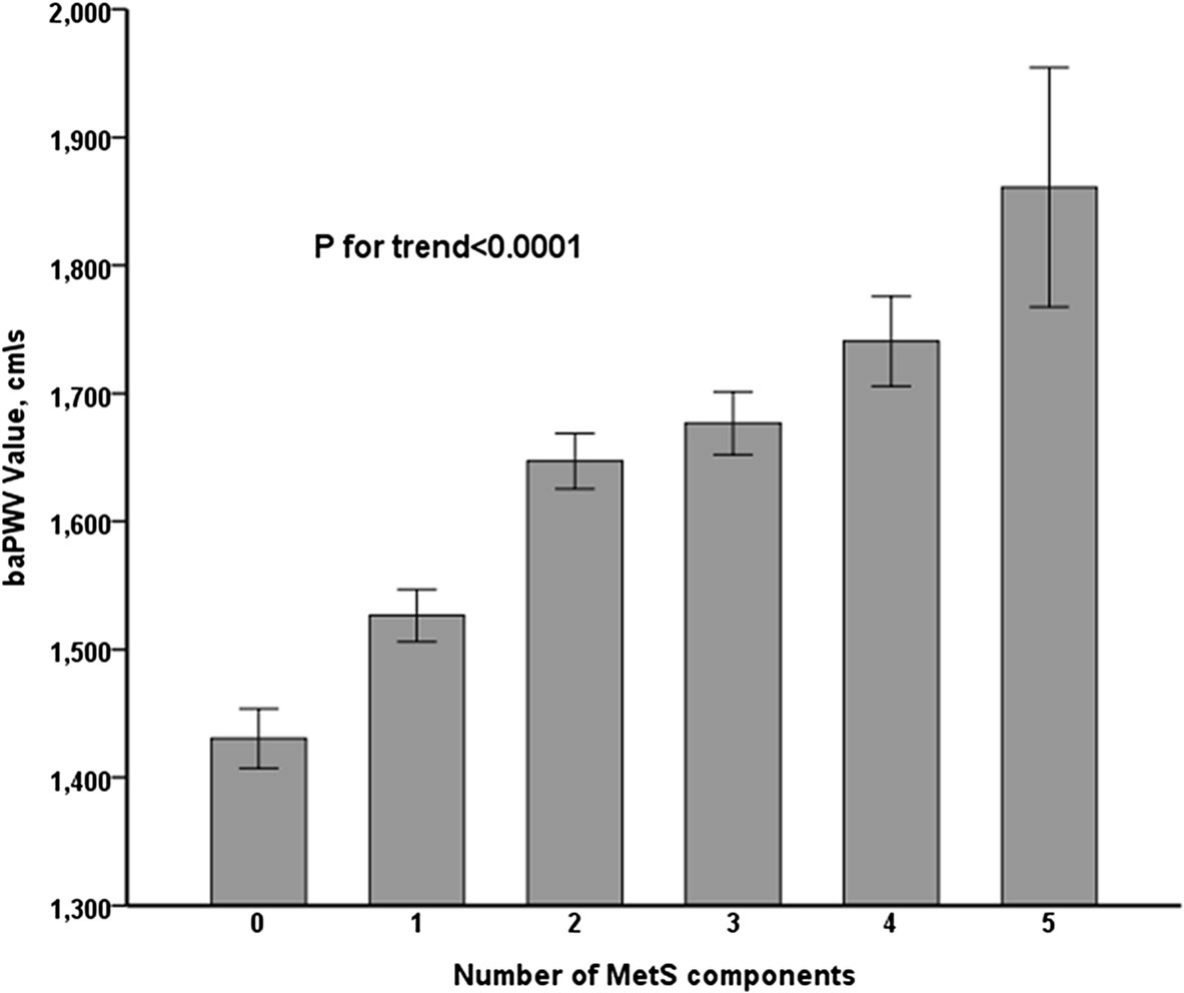
Mean baPWV according to the number (0, 1, 2, 3, 4, 5) of components of metabolic syndrome. MetS: metabolic syndrome; baPWV: brachial ankle pulse wave velocity

Mean values of baPWV in subjects with 0, 1, 2,3, 4, and 5 features of MetS were 1430, 1526, 1647, 1676,1740, and 1860 cm/s, respectively (*p* < 0.001 for trend) (Fig. 1).

The characteristics of the participants in relation to baPWV are shown in Table 2. There were significant differences between the two groups in mean age and sex (*p* < 0.0001 for both). The percentage of MetS and its components was higher in the arterial stiffness group (baPWV ≥1400 cm/s) than the non-arterial stiffness group (baPWV <1400 cm/s), and a higher proportion of subjects in the arterial stiffness group than those in the non-arterial stiffness group had exceeded thresholds for BMI, waist circumference, waist-to-hip ratio, systolic BP, diastolic BP, fasting plasma glucose, total cholesterol, triglycerides, HDL-C, LDL-C, and high-sensitivity C-reactive protein (*p* < 0.0001 for all). There was a trend toward higher age, education, hypertension, diabetes mellitus, smoking, alcohol abuse, and less or no physical activity in the arterial stiffness group than in the non-arterial stiffness group (*p* < 0.0001 for all). The severity of central obesity, elevated arterial BP, raised fasting plasma glucose and TG, and reduced HDL-C concentration and the degree of MetS were significantly higher in the arterial stiffness group than in the group without arterial stiffness.

**Table 2:** Clinical and biochemical characteristics of baPWV

Variables	baPWV < 1400cm/s (*n* = 1965)	baPWV ≥ 1400cm/s (*n* = 3216)	*P* value
Age (year)	48.14 ± 6.39	59.45 ± 12.28	<0.0001
Male, n	944 (48.04)	2164 (67.29)	<0.0001
Education, n (%)			
Illiteracy/Primary School	97 (4.94)	541 (16.82)	<0.0001
Middle School	856 (43.56)	1439 (44.75)	
High School or Higher	1012 (51.50)	1236 (38.43)	
Income, n (%)			
< ¥1000	530 (26.97)	709 (22.05)	<0.0001
¥1000–3000	1278 (65.04)	2153 (66.95)	
> ¥3000	157 (7.99)	354 (11.01)	
Body Mass Index (kg/m2)	24.05 ± 3.09	25.12 ± 3.34	<0.0001
Waist circumference (cm)	83.36 ± 9.55	87.80 ± 9.33	<0.0001
Waist-to hip ratio	0.86 ± 0.06	0.89 ± 0.06	<0.0001
Systolic Blood Pressure (mmHg)	118.78 ± 14.16	138.99 ± 19.22	<0.0001
Diastolic Blood Pressure (mmHg)	79.11 ± 9.62	85.15 ± 11.25	<0.0001
Fasting Plasma Glucose (mmol/L)	5.24 ± 0.96	5.80 ± 1.73	<0.0001
Total cholesterol (mmol/L)	1.42 ± 0.64	1.60 ± 0.73	<0.0001
Triglycerides (mmol/L)	1.50 ± 1.25	1.78 ± 1.50	<0.0001
High-Density Lipoprotein-Cholesterol (mmol/L)	1.67 ± 0.47	1.60 ± 0.44	<0.0001
Low-Density Lipoprotein-Cholesterol (mmol/L)	2.54 ± 0.66	2.68 ± 0.78	<0.0001
High-Sensitivity C-Reactive Protein	1.51 ± 2.83	2.51 ± 4.79	<0.0001
Hypertension, n (%)	427 (21.73)	2073 (64.46)	<0.0001
Diabetes mellitus, n (%)	94 (4.78)	535 (16.64)	<0.0001
Smoking, n (%)			
Never	1334 (67.89)	1899 (59.05)	<0.0001
Former	59 (3.00)	232 (7.21)	
Current	572 (29.11)	1085 (33.74)	
Alcohol abuse, n (%)			
Never	1401 (71.30)	1993 (61.97)	<0.0001
Former	18 (0.92)	63 (1.96)	
Current	546 (27.79)	1160 (36.07)	
Physical Activity, n (%)			
Inactive	877 (44.63)	1210 (37.62)	<0.0001
Moderately Active	561 (28.55)	751 (23.35)	
Very Active	527 (26.82)	1255 (39.02)	
Metabolic Syndrome, n (%)	261 (13.28)	1010 (31.41)	<0.0001
Metabolic Syndrome Components			
Central Obesity, n (%)	907 (46.16)	1838 (57.15)	<0.0001
Raised Triglycerides, n (%)	523 (26.62)	1152 (35.82)	<0.0001
Reduced High-Density Lipoprotein-Cholesterol, n (%)	169 (8.60)	419 (13.03)	<0.0001
Raised Blood Pressure, n (%)	378 (19.24)	1820 (56.59)	<0.0001
Raised Fasting Plasma Glucose, n (%)	455 (23.16)	1267 (39.40)	<0.0001
Metabolic Syndrome (No. of Components)			
0, n (%)	608 (30.94)	394 (12.25)	<0.0001
1, n (%)	644 (32.77)	751 (23.35)	
2, n (%)	427 (21.73)	959 (29.82)	
3, n (%)	216 (10.99)	681 (21.18)	
4, n (%)	64 (3.26)	371 (11.54)	
5, n (%)	6 (0.31)	60 (1.87)	

*baPWV* brachial ankle pulse wave velocity

In all four models of the multivariable logistic regression analysis, the risk of arterial stiffness increased significantly (*p* < 0.0001) with increasing number of MetS components (Table 3). After adjustment for age, gender, level of education, income, smoking, alcohol abuse, amount of physical activity, BMI, and serum concentrations of LDL-C, high-sensitivity C-reactive protein (Model 4), MetS remained significantly associated with baPWV (OR: 2.74; 95% CI: 2.28–3.30). Among the five components of MetS, raised BP was the most important factor for baPWV.

**Table 3:** Odds ratio and 95% CI of baPWV for MetS/components in multiple logistic regression analysis

Variables	Model 1	Model 2	Model 3	Model 4
Metabolic Syndrome	2.99 (2.57–3.47)	2.99 (2.54–3.53)	2.93 (2.48–3.46)	2.74 (2.28–3.30)
Central Obesity	1.10 (0.97–1.25)	1.11 (0.96–1.29)	1.08 (0.93–1.26)	1.18 (0.98–1.42)
Raised Triglycerides	1.12 (0.98–1.29)	1.36 (1.16–1.59)	1.36 (1.17–1.60)	1.38 (1.17–1.62)
Reduced High-Density Lipoprotein Cholesterol	1.46 (1.19–1.79)	1.39 (1.10–1.76)	1.40 (1.10–1.78)	1.48 (1.17–1.89)
Raised Blood Pressure	4.99 (4.36–5.71)	4.51 (3.88–5.25)	4.44 (3.82–5.18)	4.60 (3.93–5.39)
Raised Fasting Plasma Glucose	1.76 (1.54–2.01)	1.60 (1.38–1.14)	1.58 (1.35–1.84)	1.50 (1.28–1.76)
No. of Components				
0	Reference	Reference	Reference	Reference
1	1.80 (1.53–2.12)	1.89 (1.55–2.31)	1.87 (1.53–2.29)	1.97 (1.60–2.43)
2	3.47 (2.92–4.11)	3.51 (2.86–4.30)	3.38 (2.76–4.15)	3.80 (3.04–4.75)
3	4.86 (3.99–5.94)	4.98 (3.96–6.27)	4.80 (3.81–6.05)	5.70 (4.38–7.42)
4	8.94 (6.67–12.00)	9.82 (7.11–13.56)	9.39 (6.79–12.98)	11.66 (8.16–16.67)
5	15.43 (6.60–36.06)	15.80 (6.51–38.32)	15.35 (6.30–37.38)	20.88 (8.37–52.04)
P for trend	<0.0001	<0.0001	<0.0001	<0.0001

*MetS* metabolic syndrome, *baPWV* brachial ankle pulse wave velocity

Model 1: unadjusted

Model 2: adjusted for age and gender

Model 3: adjusted for age, gender, level of education, income, smoking, alcohol abuse, and amount of physical activity

Model 4: adjusted for age, gender, level of education, income, smoking, alcohol abuse, amount of physical activity, body mass index, and serum concentrations of low-density lipoprotein cholesterol, high-sensitivity C-reactive protein

The OR values were 4.60 for raised BP, 1.50 for raised fasting plasma glucose, 1.48 for reduced HDL-C, 1.38 for raised triglycerides, and 1.18 for central obesity. Using the subgroup with no component of MetS as baseline, the ORs for the associations between the subgroups with 1, 2, 3, 4 and 5 MetS components and baPWV were 1.97 (95% CI: 1.60, 2.43), 3.80 (95% CI: 3.04, 4.75), 5.70 (95% CI: 4.38, 7.42), 11.66 (95% CI: 8.16, 16.67), and 20.88 (95% CI: 8.37, 52.04), respectively. In this multivariate model, the baPWV increased significantly (*p* < 0.0001 for trend) with the number of MetS components. The same held true for the three other models of the multivariate analysis (Table 3).

**Fig. 2:**
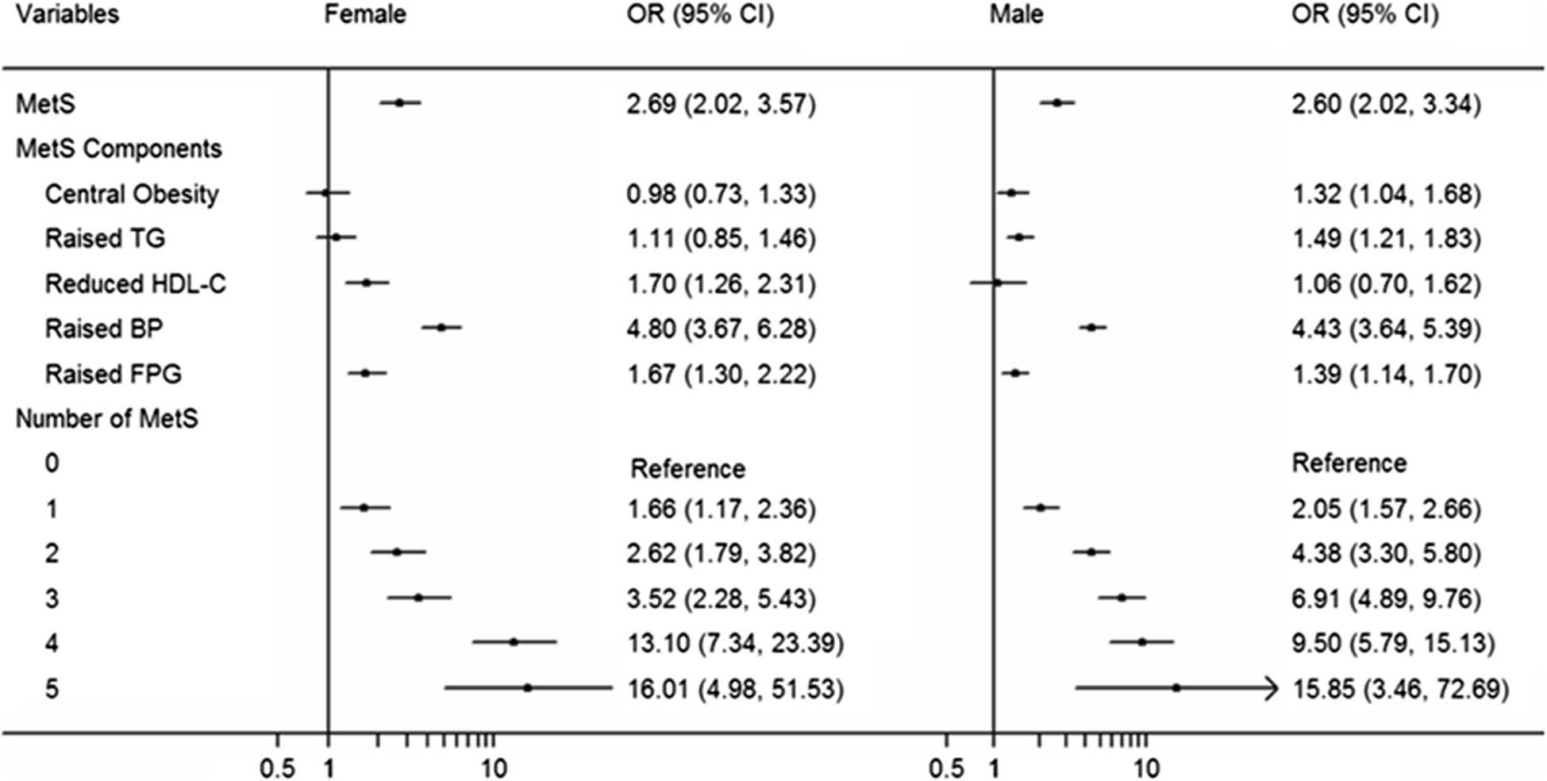
Odds ratio and 95% CI of baPWV for MetS/its Components in Multiple Logistic Regression Analysis in different gradation of gender after adjusted. MetS: metabolic syndrome; baPWV: brachial ankle pulse wave velocity; TG: triglyceride; HDL-C: high-density lipoprotein cholesterol; BP: blood pressure; FPG: fasting plasma glucose

Figure 2 shows the results of multiple logistic regression analysis of the association between MetS components and baPWV in males and females (Model 4). In this model, metabolic syndrome and its components were associated with increased baPWV in both males and females. Among the five components of MetS, raised BP was still the most important factor for baPWV. Furthermore, regardless of gender, all models of the multivariate linear regression analysis in Table 4 showed a significant positive correlation between baPWV and MetS (*p* < 0.0001).

**Table 4:** Multivariate linear regression analysis models of associations between number of MetS components and the level of baPWV

Variables		MetS		Increasing 1 component of MetS	
		B (95%CI)	*P* Value	B (95%CI)	*P* Value
	Model 1	152.24 (127.16–177.32)	<0.0001	81.21 (72.82–89.60)	<0.0001
	Model 2	125.39 (106.03–144.75)	<0.0001	64.40 (57.92–70.88)	<0.0001
	Model 3	122.17 (102.80–141.53)	<0.0001	63.07 (56.57–69.58)	<0.0001
	Model 4	132.74 (111.13–154.35)	<0.0001	77.71 (70.12–85.31)	<0.0001
Female					
	Model 1	267.94 (235.39–300.50)	<0.0001	118.25 (107.83–128.66)	<0.0001
	Model 2	129.63 (104.40–154.86)	<0.0001	59.27 (50.69–67.85)	<0.0001
	Model 3	125.63 (100.30–150.95)	<0.0001	58.03 (49.38–66.68)	<0.0001
	Model 4	127.85 (100.20–155.50)	<0.0001	66.78 (56.80–76.76)	<0.0001
Male					
	Model 1	82.90 (47.96–117.83)	<0.0001	52.95 (41.01–64.89)	<0.0001
	Model 2	107.28 (79.16–135.40)	<0.0001	63.80 (54.27–73.32)	<0.0001
	Model 3	105.18 (77.09–133.26)	<0.0001	62.51 (52.97–72.05)	<0.0001
	Model 4	126.18 (94.48–157.89)	<0.0001	81.93 (70.80–93.06)	<0.0001

*MetS* metabolic syndrome, *baPWV* brachial ankle pulse wave velocity

Model 1: unadjusted

Model 2: adjusted for age and gender

Model 3: adjusted for age, gender, level of education, income, smoking, alcohol abuse, and amount of physical activity

Model 4: adjusted for age, gender, level of education, income, smoking, alcohol abuse, amount of physical activity, body mass index, and serum concentrations of low-density lipoprotein cholesterol, high-sensitivity C-reactive protein

## Discussion

In our investigation based on a community population in northern China, baPWV was significantly associated with MetS and baPWV, and there was greater statistical significance with increasing numbers of MetS components. Among the conditions of central obesity, raised triglycerides, reduced HDL-C, raised BP, and raised fasting plasma glucose; raised BP was the strongest determinant for baPWV. Furthermore, the mean velocity of baPWV in subjects with five components of MetS had a 1.3 times faster than individuals with no MetS.

Our findings demonstrate that components of MetS for the influence of vasculature appear to be additive. This result was consistent with those of other studies [5, 10, 18]. The individuals with more risk factors have substantially greater baPWV values than those with fewer risk factors, which can be explained by baPWV being closely associated with severity of arterial stiffness.

In a Japanese population study, Nakanishi et al. [5] showed that age-adjusted mean values of baPWV were increased with clustered features of MetS in both sexes. In addition, insulin resistance was closely associated with the risk for increased baPWV or increased arterial stiffness. However, in our study, raised BP was the strongest determinant for risk of increased baPWV. However, the mechanism by which MetS might increase baPWV remains unclear. One probable explanation for our findings is that elevated BP can increase baPWV by directly acting on the arterial wall.

Angelo Scuteri et al. [11] conducted a study involving nine cohorts representing eight different European countries and the United States and suggested that the “risky” cluster of raised triglycerides, raised BP, central obesity, raised fasting plasma glucose, and central obesity was significantly associated with extremely stiff arteries. They also showed the highest prevalence in Sardinian, Italian, British, and Belgian cohorts, and the lowest in cohorts from Sweden and the United States. However, Young-Kwon Kim [18] found that the other individual MetS components, except for a raised BP, do not affect arterial stiffness independently.

Another study [19] including 8599 subjects in south China suggested that baPWV was significantly higher in subjects with MetS than in those without MetS (*p* < 0.001 for both sexes). All the metabolic components were correlated to baPWV in the male and female subjects except low HDL-C and high urine acid in the male group. BP and fasting plasma glucose had the strongest correlation factors. The baPWV values were positively correlated with advanced age (*p* < 0.001) and the values of the MetS components, and this correlation was stronger in females than in males (*p* < 0.001), which is different from the results summarized by Wang et al. [20].

Although the current study focused on the feasibility of a non-invasive baPWV examination as an effective tool to screen community populations at high risk of MetS, appropriate cut-off points or valid intervals of baPWV were not achieved [20–23]. With the marked growth of living standards in China, prevalence of MetS has increased and has resulted in serious social burden [24, 25]. Arterial stiffness is an indicator of arterial damage and its increase with age has been demonstrated to be associated with an increased risk of cardio-cerebrovascular diseases [26–29]. The clinical implication of our study is that community populations with baPWV >1400 cm/s, advanced age, and MetS with reduced HDL-C, raised BP and raised fasting plasma glucose, might benefit from immediate intervention strategies which reduce the risk of further cardiovascular disease morbidity and mortality [30].

There are some potential limitations in our study. First, our community-based investigation, which is a cross-sectional investigation, cannot prove a causal relationship, since this has to be demonstrated in a longitudinal study. The data from this study only allows us to summarize the relationships between baPWV and MetS components, while the influence of MetS as a risk factor for cardio-cerebrovascular diseases may be shown in follow-up studies. Second, this study was based on the participants of the large Kailuan Study which included employees, retirees and their families of the Kailuan Company. Although we used a rigid random sampling method to reduce selection bias, the population of the Kailuan Company does not necessarily represent the populations of Hebei province. Finally, a crucial topic is the alteration of the results of the baPWV measurements by BP levels themselves.

## Conclusions

In conclusion, our study is based on more than 5000 subjects, showing that increasing the number of MetS components is significantly correlated with a risk for increased baPWV in a northern China community-based population. A decrease in arterial compliance or an increase in arterial stiffness was shown to be related to a risk for cardiovascular mortality, for this reason, individuals with clustered components of MetS might benefit from immediate interventions, including frequent exercise, reasonable diet regulation, and medical therapy for MetS. Furthermore, baPWV may be taken into consideration as a screening index in community-based healthcare plans and as a tool for evaluating the risk of cardio-cerebrovascular diseases in clinical practice.

## Abbreviations

APAC: Asymptomatic polyvascular abnormalities community
baPWV: brachial-ankle pulse wave velocity
BMI: Body mass index
BP: Blood pressure
CI: Confidence internals
HDL-C: High-density lipoprotein cholesterol
LDL-C: Low-density lipoprotein cholesterol
MetS: Metabolic syndrome
OR: Odds ratio
SD: Standard deviation
TG: Triglyceride
